# Anticipation of Negative Pictures Enhances the P2 and P3 in Their Later Recognition

**DOI:** 10.3389/fnhum.2015.00646

**Published:** 2015-11-30

**Authors:** Huiyan Lin, Jing Xiang, Saili Li, Jiafeng Liang, Hua Jin

**Affiliations:** ^1^Key Research Base of Humanities and Social Sciences of the Ministry of Education, Center of Cooperative Innovation for Assessment and Promotion of National Mental Health, Academy of Psychology and Behavior, Tianjin Normal UniversityTianjin, China; ^2^Institute of Medical Psychology and Systems Neuroscience, University of MuensterMuenster, Germany; ^3^Shaxi Primary SchoolShenzhen, China; ^4^Center for Studies of Psychological Application, School of Psychology, South China Normal UniversityGuangzhou, China; ^5^School of Education, Guangdong University of EducationGuangzhou, China

**Keywords:** anticipation, negative pictures, recognition, ERPs, P2, P3

## Abstract

Anticipation of emotional pictures has been found to be relevant to the encoding of the pictures as well as their later recognition performance. However, it is as yet unknown whether anticipation modulates neural activity in the later recognition of emotional pictures. To address this issue, participants in the present study were asked to view emotional (negative or neutral) pictures. The picture was preceded by a cue which indicated the emotional content of the picture in half of the trials (the anticipated condition) and without any cues in the other half (the unanticipated condition). Subsequently, participants had to perform an unexpected old/new recognition task in which old and novel pictures were presented without any preceding cues. Electroencephalography data was recorded during the recognition phase. Event-related potential results showed that for negative pictures, P2 and P3 amplitudes were larger in the anticipated as compared to the unanticipated condition; whereas this anticipation effect was not shown for neutral pictures. The present findings suggest that anticipation of negative pictures may enhance neural activity in their later recognition.

## Introduction

The ability to anticipate the emotional content of an upcoming event according to environmental cues is critical in human survival, as anticipation may help individuals in preparing adaptive reactions to modulate potentially threatening situations. The modulation of anticipation on emotional events has been investigated by several electroencephalography (EEG) studies (Onoda et al., 2006, 2007; Lin et al., 2012, 2014). For example, Onoda et al. (2007) showed that the event-related desynchronization (ERD)/synchronization (ERS) was larger for negative pictures which emotional content was indicated by a preceding cue (the anticipated condition) as compared to these pictures that were presented without any cues (the unanticipated condition). Using a similar paradigm, our previous study found larger event-related potentials [ERPs; P2, N2, and late positive potential (LPP)] to emotional (positive and negative) pictures in the anticipated as compared to the unanticipated condition (Lin et al., 2012). These anticipation effects were suggested to be the result of mobilization of attention, which may enhance the encoding of the pictures.

Meanwhile, several functional magnetic resonance imaging (fMRI) and ERP studies found that anticipation of emotional pictures was relevant to their later recognition (Mackiewicz et al., 2006; Galli et al., 2011, 2014). In terms of fMRI studies, for example, the activation in the bilateral dorsal amygdala and the anterior hippocampus during anticipation of negative pictures was positively correlated to the later recognition performance of the pictures (Mackiewicz et al., 2006). For ERP studies, ERP amplitudes (200-1900 ms) during anticipation of negative pictures were more positive when the pictures were later remembered than when they were forgotten. The enhanced positivity during this time range is thought to reflect an attention bias toward threatening information. However, these effects were shown only in females (Galli et al., 2011, 2014).

The above-mentioned fMRI and ERP studies suggest that anticipation of emotional pictures is related to their later recognition performance. However, no study – to the best of our knowledge – has as yet investigated whether anticipation is relevant to neural activity during the recognition of the pictures. Therefore, we aimed to investigate this issue in the present study. While it is wise to develop the present study according to Mackiewicz et al.’s (2006) and Galli et al. ’s (2011, 2014) study, following these studies seems to make our study become complicated. For instance, Galli et al. (2011, 2014) performed repeated measures analyses of variance (ANOVAs) with recognition performance (remember vs. forgotten) and emotion (and some other factors which are unrelated to our study; i.e., emotional regulation) for ERPs during anticipation, in order to investigate the relationships between anticipation and recognition performance. If our study was investigated following Galli et al. ’s (2011, 2014) studies, we should perform ANOVAs with “neural activity in the recognition of pictures” and emotion. As the value of neural activity in the recognition of pictures should be differential for each trial; the number of levels of the factor “neural activity in the recognition of pictures” should be very large, which makes the statistical analysis become complicated.

Therefore, another approach should be used to investigate the issue of our interest. The studies shown in Paragraph 1 (Onoda et al., 2006, 2007; Lin et al., 2012, 2014) have indicated that neural activity during the encoding of emotional pictures is differential in the anticipated as compared to the unanticipated condition. Encoding, which is thought to be the first stage of memory, may influence later memory stages, such as recognition (e.g., Valentine and Bruce, 1986; Erk et al., 2005; Marzi and Viggiano, 2010). Accordingly, it is possible that anticipated as compared to unanticipated emotional pictures differ in neural activity during the recognition phase.

Therefore, the present study aimed to investigate whether anticipation of emotional pictures modulates neural activity in their later recognition. To address this issue, participants had to view emotional (negative or neutral) pictures that were presented after a cue in half of the trials and without any preceding cues in the other half. The cue indicated a specific emotion of the following picture. Subsequently, participants were asked to solve an unexpected old/new recognition task, in which all (old and novel) pictures were presented without preceding cues. Due to the high temporal resolution, ERPs were used to investigate neural activity to emotional pictures during the recognition phase. Therefore, EEG was recorded during the recognition phase. In addition, as previous studies indicate that the effect of anticipation on the recognition of emotional pictures is evident only for females but not for males (Galli et al., 2011, 2014), only female participants were recruited in the present study.

In ERP studies, the P3 (often overlapping with LPP), a positive deflection starting around 300 ms after stimulus onset over parietal scalp sites, is often associated with memory processes (e.g., Rugg et al., 1998; Curran and Cleary, 2003; Marzi and Viggiano, 2010). This P3 is supposed to reflect the allocation of attentional resources, with enhanced P3 amplitudes for the attended stimuli (e.g., Schupp et al., 2003; Kissler et al., 2009). The P3 during stimulus recognition was also suggested to be enhanced when participants had attended to the stimulus during the preceding encoding phase (e.g., Morgan et al., 2008; Langeslag et al., 2009). More importantly, Weymar et al. (2013) indicated that emotional anticipation is associated with the P3 component during the recognition phase. In this study, words (cues) were presented in two colors in order to indicate whether the following stimulus was threatening or not. Subsequently, participants were asked to recognize these words that were presented in the same color. During the recognition phase, the P3 was increased for the words which color had previously indicated the threat of the following stimulus. The authors suggested that the enhanced P3 was due to the increased attention toward the words signaling threat during the encoding phase. While the present study was to investigate the recognition of the pictures (outcomes) instead of the words (cues), the attention toward emotional pictures has been found to be enhanced by the preceding cues indicating the following threat during the encoding phase (Onoda et al., 2007; Lin et al., 2012, 2014). Therefore, we expected that anticipated as compared to unanticipated emotional pictures would evoke greater P3 amplitudes during the recognition phase.

In addition, the P2 is a positive ERP component over inferior temporal-parietal scalp sites around 180 ms after stimulus onset. Although not many studies reported the relationships between the P2 and memory; in the research field of face recognition, several studies showed that the P2 is associated with the processing of stimulus features (e.g., Stahl et al., 2008; Schulz et al., 2012a,b; Kaufmann et al., 2013). More importantly, the P2 was found to be larger in amplitude when participants recognized deeply compared to shallowly encoded faces (Marzi and Viggiano, 2010). However, it is still unclear whether this P2 effect can be shown for non-facial pictures. While most of the stimuli used in the present study would be non-facial pictures (about 95%); considering that anticipation of non-facial emotional pictures is suggested to enhance the attention toward the picture and the encoding as a result (Onoda et al., 2006, 2007; Lin et al., 2012, 2014), we expected that the P2 amplitudes would be larger for anticipated compared to unanticipated emotional pictures in the present study.

## Materials and Methods

### Participants

Twenty-four undergraduate and postgraduate students were recruited in South China Normal University via advertisement and were paid 50 RMB for participation. Four participants were excluded from analyses due to insufficient trial number for ERP averaging (*N* < 20), resulting in a total of 20 participants (all females; 20–25 years old, *M* = 22.06, *SD* = 1.39). All participants were right-handed as determined by the Edinburgh Handedness Inventory (Oldfield, 1971). Participants had normal or corrected-to-normal vision and none of the participants reported a history of neurological illness. The study was conducted in accordance with standard ethical guidelines as defined in the Declaration of Helsinki and written informed consent was obtained prior to the testing. This study was approved by the ethics committee of School of Psychology, South China Normal University.

### Stimuli

Stimuli were 480 colored pictures (240 negative and 240 neutral). They were obtained from various sources, including the International Affective Picture System (IAPS; Lang et al., 2008), Chinese Affective Picture System (CAPS; Bai et al., 2005) and public pictures available on the internet. With respect to these pictures, contents were varied, such as animals, scene, objects, people and buildings. For each category of contents, the number of negative and neutral pictures was similar. For all the pictures, we adjusted the size to 11 cm × 9 cm (horizontal × vertical), converted the pictures to gray-level, and matched the pictures in luminance and contrast (e.g., the value of luminance and contrast is the same for all pictures) with the help of Adobe Photoshop CS6.

The pictures were rated on valence and arousal using a 9-point scale ranging from “1” (extremely unpleasant) to “9” (extremely pleasant) and “1” (low arousal) to “9” (high arousal), respectively, by another group of 109 undergraduate and postgraduate students (all females, 19–27 years old, *M* = 21.68, *SD* = 1.59). The ratings were higher for neutral as compared to negative pictures in valence [neutral vs. negative: 4.94 ± 0.06 (*M ± SE*) vs. 2.64 ± 0.08; *F*(1,108) = 686.23, *p* < 0.001, $\eta_{p}^{2}$ = 0.86] but lower in arousal [neutral vs. negative: 2.88 ± 0.13 vs. 5.09 ± 0.15; *F*(1,108) = 175.23, *p* < 0.001, $\eta_{p}^{2}$ = 0.62].

Of these pictures, half of them (120 negative and 120 neutral; old pictures) were presented during both the encoding and the recognition phase. The other half (120 negative and 120 neutral) were used as novel pictures and were presented only during the recognition phase. Old and novel pictures were similar in valence (*ps* > 0.10), arousal (*ps* > 0.10) and contents. Each emotional category of old pictures was pseudo-randomly separated into two sets according to the ratings of valence and arousal and the contents. These four sets of old pictures were used to create four experimental conditions: anticipated-negative, unanticipated-negative, anticipated-neutral and unanticipated-neutral. Assignments of sets were counterbalanced across participants.

### Procedure

After the informed consent had been given and handedness had been determined, participants were asked to sit in a quiet and dimly room at a viewing distance of approximately 1 m from a 17-inch computer screen (screen resolution 640 × 480 pixels). Presentations of stimuli and recordings of behavioral responses were controlled by E-Prime 1.1 software (Psychology Software Tools, Inc., Pittsburgh, PA, USA). All stimuli were presented against a gray background.

Prior to the encoding phase, participants were told that they would be always presented with pictures and that sometimes the picture would be preceded by a cue. Participants were also told the meaning of the cue (e.g., the symbol “#” and “^∗^” will be always followed by a negative and a neutral picture, respectively). The meanings of the cues were counterbalanced across participants. Participants were asked to view the cues and the pictures during their presentations. In order to allow participants to pay attention to the experiment, they were also asked to rate the pleasantness of the pictures on a 9-point scale ranging from “1” (extremely unpleasant) to “9” (extremely pleasant) after the pictures by using the number keypad on the keyboard. Every trial started with a black fixation cross for 500 ms, replaced by a blank screen for 600 to 1000 ms (*M* = 800 ms). For half of the trials, a black cue (“^∗^” or “#”) was presented for 200 ms, followed by a blank screen for 1600 to 2000 ms (*M* = 1800 ms). In the other half of the trials, the blank screen was presented continually, matching the duration of the cue and the blank screen following the cue. Subsequently, a picture was presented for 1000 ms. After another blank screen for 200 ms, a rating scale was presented and labeled as follows: 1 = “extremely unpleasant” to 9 = “extremely pleasant”. There was no time limit for the response. The subsequent trial started after another blank screen for 1000 ms. According to the emotion of the picture and the presence of the cue, there were four experimental conditions (anticipated-negative, unanticipated-negative, anticipated-neutral, and unanticipated-neutral). The sequence of experimental conditions was presented randomly. For each experimental condition, each picture was presented once, resulting in a total of 240 trials.

The recognition phase followed the encoding phase after approximately 10 min. Participants were informed about the recognition task only at this point in the experiment. Participants were asked to indicate whether the prompted picture had been presented in the preceding encoding phase (old pictures) or not (novel pictures) by pressing the “F” or the “J” key with the left and the right index finger, respectively. The instructions emphasized speed as well as accuracy. Response assignments were counterbalanced across participants. Each trial started with a blank fixation cross for 500 ms, followed by a blank screen for 600 to 1000 ms (*M* = 800 ms). Subsequently, either an old or a novel picture was presented for 1000 ms. The subsequent trial started after the presentation of another blank screen for 1000 ms. Button presses were allowed during the presentation of the picture or the following blank. Each picture was presented once, resulting in 480 trials in total. The complete experiment including the encoding and the recognition phase lasted about 1.5 h.

### Behavioral Recording

During the encoding phase, the ratings of pleasantness for the pictures were recorded. During the recognition phase, response times and hit rates were recorded from the onset of the picture to the offset of the following blank. As many previous studies analyzed trials with correct responses only (e.g., Keightley et al., 2011; Schulz et al., 2012a,b), response times in the present study were also analyzed for correct responses only (**Table 1**).

**Table 1:** *Mean* number of trials of response time (RT) and event-related potential (ERP) analysis, respectively, for all experimental conditions.

	Neutral		Negative	
	Anticipated	Unanticipated	Anticipated	Unanticipated
Response times	44.75	43.85	45.50	43.75
ERPs	40.90	40.75	40.45	39.90

### EEG Recording

Electroencephalography was continuously recorded using a Neuroscan Synamps2 AC-amplifier (Neuroscan, Inc., Sterling, VA, USA). The Ag/AgCl electrodes were placed on the scalp by a 32 channel Quick-Cap according to the 10–20 international system (FP1, FP2, F7, F3, Fz, F4, F8, FT7, FC3, FCz, FC4, FT8, T7, C3, Cz, C4, T8, TP7, CP3, CPz, CP4, TP6, P7, P3, Pz, P4, P8, O1, Oz, and O2). EEG electrodes were connected to ground and were referenced to the right mastoid online. The horizontal electrooculogram (EOG) was recorded from two electrodes at the outer canthi of both eyes, and the vertical EOG was recorded bipolarly from two electrodes above and below the left eye to monitor eye blinks and movements. All signals were digitized with a sampling rate of 1000 Hz/channel with a 50 Hz notch filter and were used a 0.05–100 Hz band-pass. Electrode impedances were maintained below 5 kΩ.

The EEG was analyzed oﬄine using the SCAN 4.3 software (Neuroscan, Inc., Sterling, VA, USA). Raw EEG signals were digitally re-referenced to the average of two mastoids. Ocular movements were inspected and removed from the EEG signal using a regression procedure implemented with NeuroScan 4.3 software (Semlitsch et al., 1986). EEG data were then segmented into 1100 ms epochs from –100 to 1000 ms relative to the onset of old picture during the recognition phase, with the first 100 ms epochs for baseline correction. As the neural activity has been found to be different between “remember” and “forget” stimuli (e.g., Reber et al., 2002; Hsieh et al., 2009) and many ERP studies analyzed “remember” trials only (e.g., Kaufmann et al., 2013; Weymar et al., 2013; Lin et al., 2015), the present study analyzed only the correct response old pictures. Artifact rejection was carried out using an amplitude threshold of 100 μV. Trials were averaged separately for each channel and each experimental condition. Averaged ERPs were low-pass filtered at 30 Hz (24 db/oct, Butterworth zero phase shift), and ERPs were then recalculated to average reference, excluding vertical, and horizontal EOG channels. The minimum number of trials for a participant in any experimental conditions was 20 (**Table 1**).

Event-related potentials were quantified by mean amplitudes for P2 (180–230 ms) and P3 (330–400 ms) relative to –100 ms baseline. The P2 was measures at the electrodes P7/8 and the P3 was measured at the electrode Pz. Electrodes of interest and the time windows of these components were chosen according to visual inspection of the grand waveforms and previous studies (e.g., Stahl et al., 2008; Marzi and Viggiano, 2010; Schulz et al., 2012a,b; Kaufmann et al., 2013).

It should be noted that the EEG was recorded also during the encoding phase. However, the present study would not further investigate the ERP effects of anticipation during this phase, as this issue had been investigated in our previous study (Lin et al., 2012).

### Data Analysis

For statistical analyses of behavioral data, the ratings of pleasantness for pictures during the encoding phase and response times and hit rates for old pictures during the recognition phase were entered into 2 × 2 repeated ANOVAs with anticipation (anticipated vs. unanticipated) and emotion (negative vs. neutral) as within-subject factors. For ERPs during the recognition phase, repeated measures ANOVAs with within-subject factors anticipation (anticipated vs. unanticipated) and emotion (negative vs. neutral) were performed separately for the P2 and P3. The analysis for the P2 included hemisphere (left vs. right) as an additional within-subject factor. Degrees of freedom and *p*-values of ANOVAs were corrected using Greenhouse–Geisser correction where necessary.

## Results

### Behavioral Results

#### Ratings of Pleasantness

**FIGURE 1:**
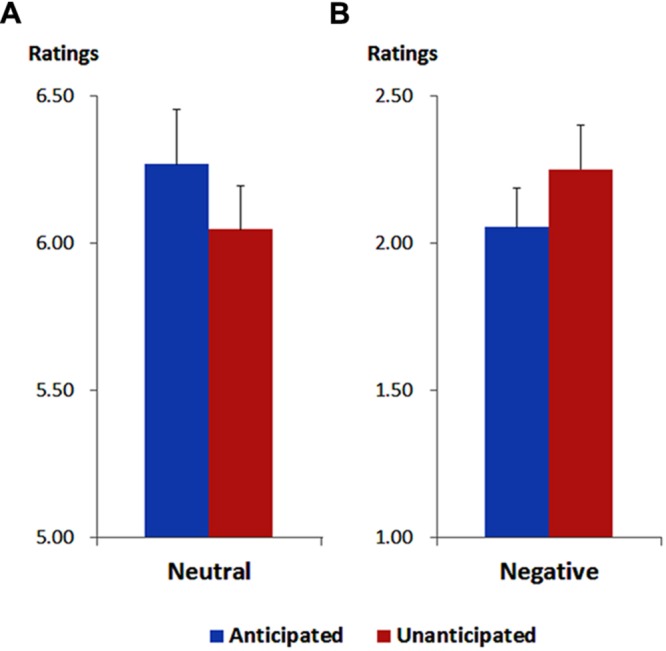
**Ratings of pleasantness for neutral **(A)** and negative **(B)** pictures in each anticipation condition.** Vertical lines indicate the standard error of the mean.

As illustrated in **Figure 1**, the ANOVA showed a main effect of emotion [*F*(1,19) = 324.88, *p* < 0.001, $\eta_{p}^{2}$ = 0.95). The ratings of pleasantness were generally higher for neutral (6.16 ± 0.16) compared to negative pictures (2.15 ± 0.14).

More importantly, the interaction between anticipation and emotion was significant [*F*(1,19) = 13.37, *p* = 0.002, $\eta_{p}^{2}$ = 0.41). For neutral pictures, the ratings were higher in the anticipated (6.27 ± 0.19) compared to the unanticipated condition [6.05 ± 0.15; *F*(1,19) = 9.44, *p* = 0.006, $\eta_{p}^{2}$ = 0.33]. For negative pictures, however, the ratings were higher in the unanticipated (2.25 ± 0.15) compared to the anticipated condition [2.05 ± 0.13; *F*(1,19) = 4.86, *p* = 0.040, $\eta_{p}^{2}$ = 0.20]. These findings are in line with our previous study (Lin et al., 2012).

#### Hit Rates and Response Times

**FIGURE 2:**
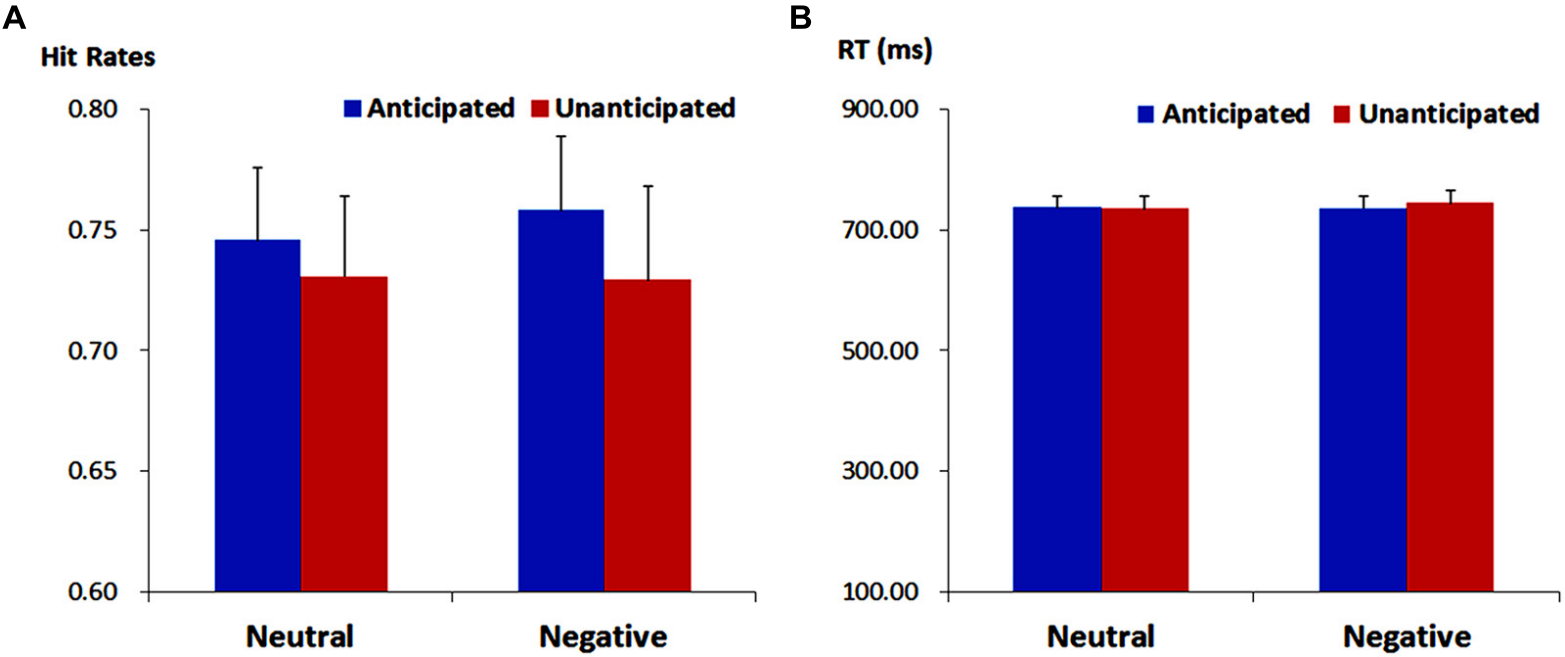
**Hit rates (ACC, **A**) and response times (RT, **B**) for pictures in each experimental condition.** Vertical lines indicate the standard error of the mean.

For response times, no main effects or interaction was significant (*ps* > 0.10). The analysis on hit rates revealed a main effect of anticipation [*F*(1,19) = 4.37, *p* = 0.050, $\eta_{p}^{2}$ = 0.19]. The hit rates were higher for anticipated (75.21 ± 2.72%) as compared to unanticipated pictures (73.00 ± 3.22%). Neither the main effect of emotion nor the interaction between anticipation and emotion reached statistical significance (*ps* > 0.10; **Figure 2**).

### ERP Results

#### P2

**FIGURE 3:**
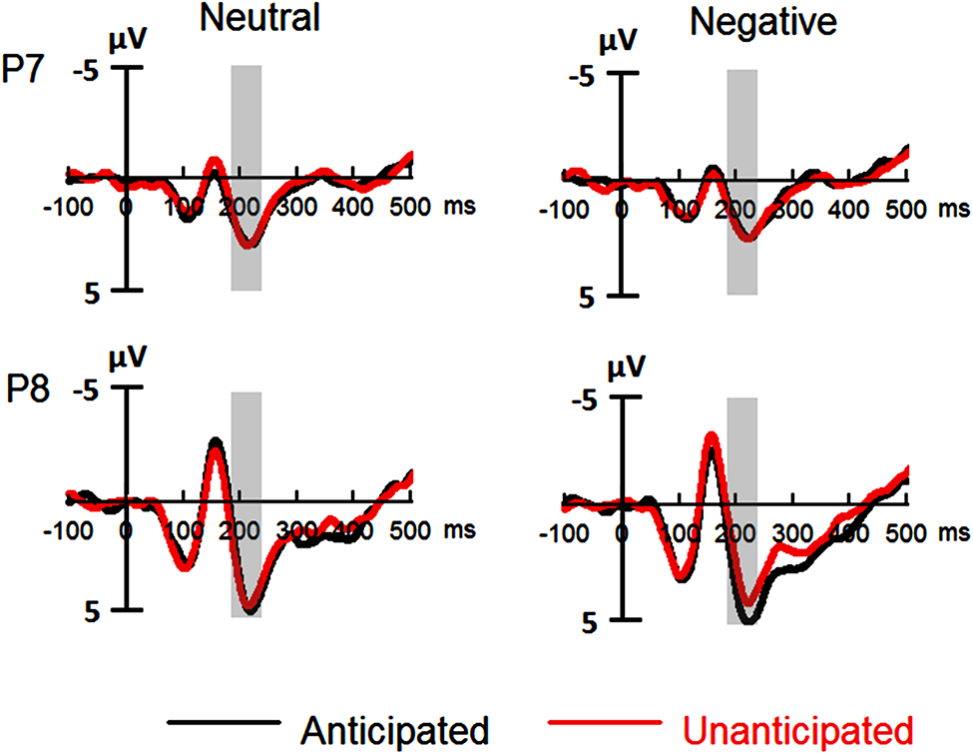
**Event-related potentials (ERPs) at P7 and P8 for all the experimental conditions.** Shaded areas correspond to the analysis window for the P2 (180–230 ms).

The analysis on P2 amplitudes revealed a three-way interaction [*F*(1,19) = 6.51, *p* = 0.020, $\eta_{p}^{2}$ = 0.26]. For neutral pictures, neither main effects of anticipation and hemisphere nor their interaction was significant (*ps* > 0.10). For negative pictures, while main effects of anticipation and hemisphere was not significant (*ps* > 0.10), there was a significant interaction between these two factors [*F*(1,19) = 7.65, *p* = 0.012, $\eta_{p}^{2}$ = 0.29]. The amplitudes were larger for anticipated (3.79 ± 1.12 μV) as compared to unanticipated negative pictures (2.82 ± 1.11 μV) over the right hemisphere [*F*(1,19) = 6.08, *p* = 0.023, $\eta_{p}^{2}$ = 0.24], but the P2 amplitudes were similar in these two conditions over the left hemisphere (*ps* > 0.10). Other main effects or interactions did not reach statistical significance (*ps* > 0.10; **Figures 3** and **5**).

#### P3

For P3 amplitudes, there was a main effect of emotion [*F*(1,19) = 32.98, *p* < 0.001, $\eta_{p}^{2}$ = 0.63]. The amplitudes were larger for negative (4.41 ± 0.83 μV) as compared to neutral pictures (2.72 ± 0.85 μV).

**FIGURE 4:**
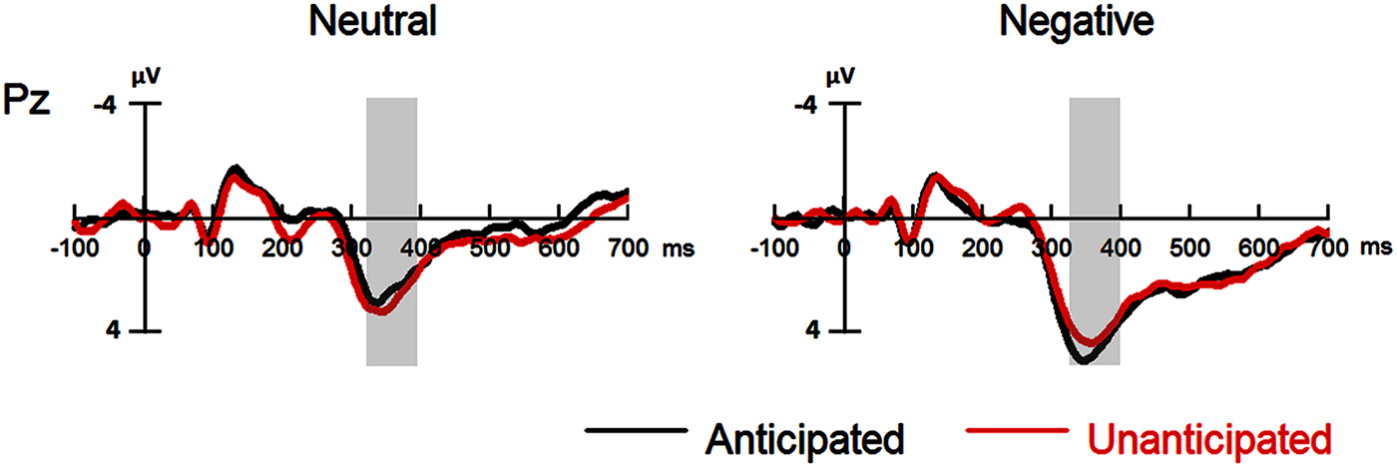
**Event-related potentials at Pz for all the experimental conditions.** Shaded areas correspond to the analysis window of the P3 (330–400 ms).

**FIGURE 5:**
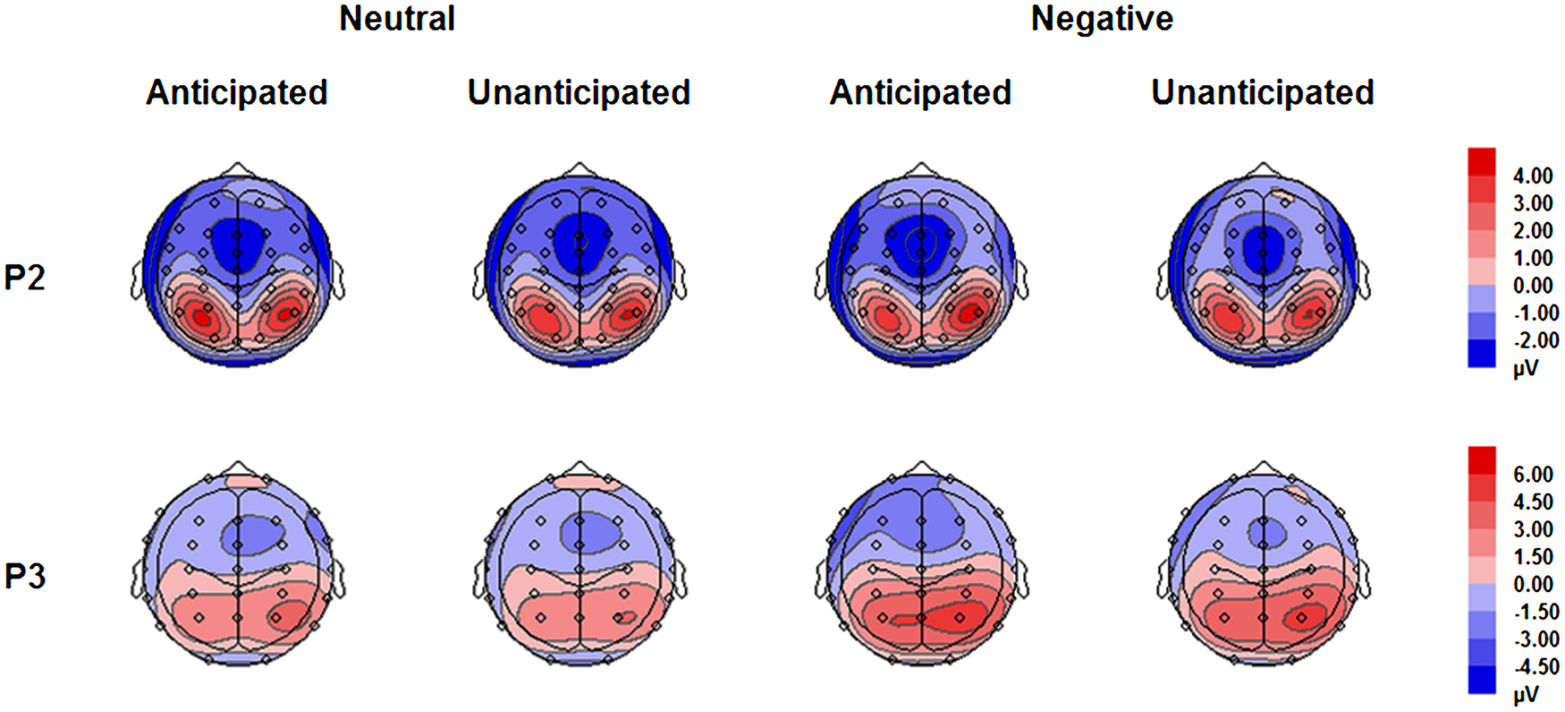
**Topographical maps based on mean amplitudes of P2 (180–230 ms) and P3 (330–400 ms) for all experimental conditions**.

The interaction between anticipation and emotion was significant [*F*(1,19) = 4.91, *p* = 0.039, $\eta_{p}^{2}$ = 0.21). For negative pictures, the amplitudes were larger in the anticipated (4.74 ± 0.83 μV) as compared to the unanticipated condition [4.08 ± 0.86 μV; *F*(1,19) = 4.97, *p* = 0.038, $\eta_{p}^{2}$ = 0.21]; for neutral pictures, however, the effect of anticipation did not reach statistical significance (*ps* > 0.10). Other main effects or interactions did not reach statistical significance (*ps* > 0.10; **Figures 4** and **5**).

## Discussion

The present study investigated whether anticipation of emotional (negative and neutral) pictures modulates ERPs in their later recognition. ERP results showed that for negative pictures, P2 and P3 amplitudes were larger in the anticipated as compared to the unanticipated condition; but these anticipation effects were not observed for neutral pictures. The findings indicate that anticipation of negative pictures alters neural activity in their later recognition.

The P3 is an ERP component which has been repeatedly shown to be related to memory (e.g., Rugg et al., 1998; Curran and Cleary, 2003; Marzi and Viggiano, 2010). The P3 is thought to be relevant to the allocation of attentional resources, with larger amplitudes for the attended stimuli (e.g., Schupp et al., 2003; Kissler et al., 2009). Accordingly, the present study may indicate that anticipated as compared to unanticipated negative pictures capture more attentional resources during the recognition phase. Anticipation about the emotional content of an upcoming stimulus allows individuals to “pre-process” the related emotional content before the occurrence of the stimulus in order to modulate the stimulus after (e.g., Nitschke et al., 2006). Previous EEG studies have shown that anticipation of negative pictures enhances neural (e.g., ERS/ERD and ERP) responses to the pictures, indicating that anticipation increases the attention toward negative pictures during the encoding phase (Onoda et al., 2006, 2007; Lin et al., 2012, 2014). More importantly, when participants had enhanced the attention toward the stimuli during the encoding phase, this increased attention was suggested to be shown again during the recognition phase (e.g., Morgan et al., 2008; Langeslag et al., 2009). Therefore, in the present study, anticipation of negative pictures may have enhanced the attention toward the pictures during the encoding phase and later, increase again during the recognition of the pictures even though there is no anticipation in recognition.

This finding might be in accordance with a study by Weymar et al. (2013). In this study, emotional words were presented in two colors in order to indicate whether the following stimulus was threatening or not. When participants were asked to recognize these words in a later recognition task, the P3 amplitudes were enhanced for the words which color had indicated the potential threat. The authors implied that the enhanced P3 was due to the increased attention toward the words’ color signaling threat during the encoding phase. Thus, similar mechanisms may underlie the effect of negative anticipation on P3 amplitudes during stimulus recognition in methodologically completely different studies.

Another finding in the present study is that the posterior P2 amplitudes were larger for anticipated as compared to unanticipated negative pictures. Several studies suggest that the P2 during stimulus recognition is associated with the processing of stimulus features (e.g., Stahl et al., 2008; Schulz et al., 2012a,b; Kaufmann et al., 2013). Importantly for the present study, Marzi and Viggiano (2010) showed that deeply compared to shallowly encoded faces evoked larger P2 amplitudes during face recognition. Therefore, in the present study, it is possible that anticipation enhances the attention toward negative pictures, which results in the elaborate encoding, and thus, is facilitated in the processing of features during the later recognition of the pictures.

Different from negative pictures, we did not find that anticipation modulated the P2 and P3 during recognition of neutral pictures, implying that anticipated compared to unanticipated neutral pictures are similar in the processing of features and in the allocation of attentional resources during the recognition phase (e.g., Morgan et al., 2008; Langeslag et al., 2009; Marzi and Viggiano, 2010). Previous studies suggest that anticipation enhances the attention toward negative pictures during the encoding phase; this anticipation effect is less sensitive for non-negative pictures (Onoda et al., 2006, 2007). Therefore, in the present study, the anticipation effect on neutral pictures may also be not very strong during the encoding phase, which results in failing to observe the anticipation ERP effects during the recognition phase.

For the behavioral data, hit rates were generally higher in the anticipated as compared to the unanticipated pictures, regardless of emotion. This effect may be due to the enhanced attention toward emotionally anticipated pictures during the encoding phase (Onoda et al., 2006, 2007; Lin et al., 2012, 2014). However, previous studies did not show any relationships between anticipation and behavioral performance of neutral pictures (Mackiewicz et al., 2006; Galli et al., 2011, 2014). Therefore, it seems to be too early to make a conclusion that anticipation enhances the recognition performance of neutral pictures. In future studies, we hope to investigate this issue in more detail.

In addition, we did not find any effects of response times. The reason may be that response times are not a sensitive and an important index in long-term memory related to emotional stimuli. Many previous studies did not find the effects of response times and some even did not report their results (e.g., Dolcos et al., 2004; Sharot et al., 2004; Anderson et al., 2006; Satterthwaite et al., 2009). Therefore, it is not surprising that there were no effects of response times in the present study.

While the present study found that anticipation of emotional pictures modulated the ERPs in the later recognition of the pictures, these results were obtained from a recognition task which was performed immediately after the encoding phase. Mackiewicz et al. (2006) found that while anticipation of emotional pictures was correlated with their later recognition performance in the immediate recognition task, this was not the case when the recognition task was performed 2 weeks after the encoding phase. Therefore, it is still unclear whether the ERP effects shown in the present study will still be evident in a delayed recognition task. Future studies may be devoted to investigate this issue in more details. In addition, the present study did not investigate the anticipation ERP effects related to recognition for positive pictures. Our previous studies showed that anticipation enhanced the encoding of positive pictures (Lin et al., 2012, 2014). Therefore, it is possible that the anticipation ERP effects related to recognition shown in the present study will be also observed for positive pictures. In future studies, we may further investigate this issue in more detail. Moreover, we may also use negative and positive pictures with high and low arousal to further investigate whether the anticipation ERP effects of recognition is related to the valence, the arousal or their interaction.

## Conclusion

Our findings revealed that anticipation of negative pictures modulated ERP responses in their later recognition. The P2 and P3 amplitudes were larger for negative pictures in the anticipated as compared to the unanticipated condition. However, these anticipation effects were absent for neutral pictures. Therefore, the findings may indicate that anticipation of negative pictures enhances neural activity in their later recognition.

## Author Contributions

HL was involved in study design, data analysis and manuscript drafting and revises. JX was involved in study design, execution, data analysis and manuscript revises. SL and JL were involved in data analysis and manuscript revises. HJ was involved in study design and manuscript revises. We have read and approved the manuscript and agree to be accountable for all aspects of the work in ensuring that questions related to the accuracy or integrity of any part of the work are appropriately investigated and resolved.

## Conflict of Interest Statement

The authors declare that the research was conducted in the absence of any commercial or financial relationships that could be construed as a potential conflict of interest.
